# Functional Connectivity in Chronic Schizophrenia: An EEG Resting‐State Study with Corrected Imaginary Phase‐Locking

**DOI:** 10.1002/brb3.70370

**Published:** 2025-03-13

**Authors:** Christophe Domingos, Wiktor Więcławski, Sandra Frycz, Maja Wojcik, Martin Jáni, Olga Dudzińska, Przemysław Adamczyk, Tomas Ros

**Affiliations:** ^1^ Institute of Psychology Jagiellonian University Krakow Poland; ^2^ Life Quality Research Centre (CIEQV) Sport Science School of Rio Maior Rio Maior Portugal; ^3^ Doctoral School in the Social Sciences Jagiellonian University Krakow Poland; ^4^ Department of Psychiatry, Faculty of Medicine Masaryk University and University Hospital Brno Brno Czech Republic; ^5^ Department of Clinical Neuroscience University of Geneva Geneva Switzerland; ^6^ Center for Biomedical Imaging (CIBM) Geneva‐Lausanne Switzerland

**Keywords:** EEG, functional connectivity, phase locking value, power spectrum, resting‐state, schizophrenia

## Abstract

Schizophrenia is a complex disorder characterized by altered brain functional connectivity, detectable during both task and resting state conditions using different neuroimaging methods. To this day, electroencephalography (EEG) studies have reported inconsistent results, showing both hyper‐ and hypo‐connectivity with diverse topographical distributions. Interpretation of these findings is complicated by volume‐conduction effects, where local brain activity fluctuations project simultaneously to distant scalp regions (zero‐phase lag), inducing spurious inter‐electrode correlations.

**Aim**: In the present study, we explored the network dynamics of schizophrenia using a novel functional connectivity metric—corrected imaginary phase locking value (ciPLV)—which is insensitive to changes in amplitude as well as interactions at zero‐phase lag. This method, which is less prone to volume conduction effects, provides a more reliable estimate of sensor‐space functional network connectivity in schizophrenia.

**Methods**: We employed a cross‐sectional design, utilizing resting state EEG recordings from two adult groups: individuals diagnosed with chronic schizophrenia (*n* = 30) and a control group of healthy participants (*n* = 30), all aged between 18 and 55 years old.

**Results**: Our observations revealed that schizophrenia is characterized by a prevalence of excess theta (4–8 Hz) power localized to centroparietal electrodes. This was accompanied by significant alterations in inter‐ and intra‐hemispheric functional network connectivity patterns, mainly between frontotemporal regions within the theta band and frontoparietal regions within beta/gamma bands.

**Conclusions**: Our findings suggest that patients with schizophrenia demonstrate long‐range electrophysiological connectivity abnormalities that are independent of spectral power (i.e., volume conduction). Overall, distinct hemispheric differences were present in frontotemporo‐parietal networks in theta and beta/gamma bands. While preliminary, these alterations could be promising new candidate biomarkers of chronic schizophrenia.

## Graphical abstract

Using ciPLV, an EEG connectivity metric less affected by volume conduction, we found that schizophrenia patients exhibit distinct long‐range functional connectivity abnormalities in theta and beta/gamma bands—suggesting new potential biomarkers for chronic schizophrenia.

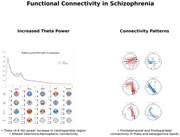

## 1 Introduction

Schizophrenia (SCZ) is a debilitating mental disorder characterized by a wide spectrum of symptoms in affected individuals and affects about 1% of the total global population (McGrath et al. 2008; van Os and Kapur 2009). These symptoms are most often classified into positive (e.g., hallucinations, delusions), negative (e.g., anhedonia, avolition), and disorganized dimensions (e.g., disorganized speech and behavior) (Tandon et al. 2013; Xiao et al. 2022), along with cognitive and social impairments that are often associated with a decrease in daily functioning even when a patient is not in an active psychotic episode (Bowie et al. 2006; Schaefer et al. 2013). They are associated with problems in social integration and employment, which have a major adverse effect on quality of life.

Patients with SCZ demonstrate conspicuous behavioral differences compared to neurotypical subjects, which are accompanied by substantial abnormalities in neuroimaging data utilizing functional magnetic resonance imaging (fMRI) (Whitfield‐Gabrieli and Ford 2012) or EEG (Ford et al. 2016). Neuroimaging studies have revealed altered functional connectivity patterns, suggesting that SCZ may involve disruptions in the coordinated activity of brain networks (Fornito et al. 2015). Nonetheless, the inconsistent findings across a number of studies (Northoff and Duncan 2016) call for more research that could shed light on the neurophysiological basis of SCZ (Northoff and Qin 2011). Such data inconsistencies may thus reflect methodological differences, patient heterogeneity, and the intrinsic complexity of brain network interactions highlighting the need for more sophisticated analytical approaches.

Resting‐state EEG neuroimaging studies of SCZ have been done to investigate relative power and functional connectivity differences in an attempt to understand the disorder as potentially one of functional “disconnection” that limits the integration of mental processes (Friston and Frith 1995; Stephan et al. 2009). Functional network connectivity (FNC) —that is, the temporal correlation of activities between brain regions— is a pivotal concept in theories of neural communication (Fingelkurts et al. 2005; Fries 2015). EEG provides high temporal resolution, allowing for the investigation of oscillatory activity and synchronization between different brain regions in various frequency bands (Buzsaki and Draguhn 2004; Uhlhaas and Singer 2010).

SCZ has been shown to exhibit varying degrees of hypo‐ and hyper‐connectivity with studies suggesting a complex signature of its neural network dynamics (Chang et al. 2024; di Lorenzo et al. 2015; Hinkley et al. 2011; Kirino et al. 2017). For instance, some studies report decreased alpha‐band connectivity, which may relate to deficits in cognitive processes such as attention and memory (Hirano et al. 2015; Ranlund et al. 2014). On the contrary, there is a correlation between increased gamma‐band connectivity with positive symptoms such as hallucinations (Uhlhaas and Singer 2010). The inconsistencies across studies are consistent with the complexity of SCZ and indicate that SCZ may involve both hypo‐ and hyper‐connectivity, which may reflect aspects of different pathophysiological mechanisms or different subtypes of the disorder.

Traditional measures of functional connectivity such as coherence and the basic phase locking value (PLV) cannot protect against volume conduction and common source effects that inflate connectivity estimates from zero‐lag synchronizations (Nolte et al. 2004; Stam et al. 2007). This renders interpreting connectivity results unobservable difficult, as it makes it challenging to tell apart true neural interactions from spurious connections induced by these confounding factors. In order to address this issue, advanced connectivity metrics are developed to minimize the effects of zero‐phase lag connectivity.

To explore FNC differences of chronic SCZ, we used the latest advances in EEG signal analysis, and a new metric, called the corrected imaginary phase locking value (ciPLV) (Bruna et al. 2018). The ciPLV algorithm improves on the classical PLV by specifically removing the contribution of zero‐phase connectivity, thereby reducing biased estimates that may arise due to differences in local spectral power and volume conduction (Bruna et al. 2018). Unlike other metrics such as the imaginary part of coherency (Nolte et al. 2004), which also attempts to exclude zero‐lag components, ciPLV corrects for the bias introduced by the non‐uniform distribution of phase differences, enhancing the detection of true connectivity differences (Bruna et al. 2018). The fact that both subtle connectivity alterations are expected makes ciPLV well‐suited to studying psychiatric disorders such as SCZ.

In addition, we performed our analysis in sensor space to maintain inter‐electrode phase relations that can be affected by varying orientations of dipoles (He et al. 2011). Source‐space analyses are helpful for the spatial localization of neural generators; however, the inaccuracy of source reconstruction routines and head models may contribute to additional errors (Michel and Brunet 2019). By contrast, sensor space analysis enables one to directly examine the recorded EEG signals without these assumptions, a desirable option for analyzing connectivity patterns rather than precise localization of sources (Nunez et al. 1994). Furthermore, sensor space connectivity is more sensitive in detecting group differences within the clinical population (Mahjoory et al. 2017).

We aimed to uncover the most salient EEG abnormalities in chronic SCZ, that is, alterations in relative power within the canonical frequency bands (Cao et al. 2022; Iglesias‐Tejedor et al. 2022; Newson and Thiagarajan 2018), as well as functional connectivity using ciPLV (Bastos and Schoffelen 2015). By leveraging ciPLV, our principal hypothesis was that SCZ would demonstrate connectivity differences that would diverge (i.e., be regionally distinct) from patterns of abnormalities in spectral power. Specifically, we expected to find altered connectivity patterns that are not merely a consequence of changes in power but reflect genuine disruptions in neural communication pathways.

We aimed to discover new EEG biomarkers that reflect brain communication disturbances in SCZ by mapping functional connectivity signatures that define SCZ. Such biomarkers may greatly aid our understanding of the pathophysiology of SCZ, and they can potentially inform the development of targeted interventions (Ford et al. 2014; Uhlhaas and Singer 2012). To better understand neural alterations in SCZ, we conduct a comprehensive analysis of both spectral power and functional connectivity

We conduct a sensor space analysis in order to keep dipolar source dipolar source orientations that can alter inter‐electrode phase relations. Sensor space analysis allows us to measure connectivity patterns as they are directly measured, and this is particularly valuable when considering possible changes to the orientation and strength of neural sources in SCZ. The goal was to discover potentially new functional connectivity pathways associated with brain communication disturbances in SCZ.

## 2 Methods

### 2.1 Participants

The sample included 30 outpatients diagnosed with SCZ and 30 healthy control participants (CON), matched with respect to age and sex. All participants provided informed consent before participating in the study, which included interviews, the Montreal Cognitive Assessment (MoCA) (Nasreddine et al. 2005), resting‐state EEG recordings, and, for SCZ subjects, assessments using the Positive and Negative Syndrome Scale (PANSS) (van der Gaag et al. 2006) and the Brief Negative Symptom Scale (BNSS) (Kirkpatrick et al. 2011). Clinical interviews were conducted by two clinical psychiatrists who were trained and experienced in administering the PANSS and BNSS. To ensure consistency and reliability of the assessments, inter‐rater reliability was evaluated using Cohen's kappa statistics (Landis 1977). The kappa values were 0.85 for PANSS and 0.80 for BNSS, indicating substantial agreement between the raters. Inclusion criteria for patients were diagnosis of SCZ according to DSM‐5 criteria, age between 18 and 60 years, and stable psychopathological condition. Exclusion criteria comprised the history of head injuries or neurological disorders (e.g., seizures), substance dependence or abuse within the past six months, any current somatic illnesses affecting brain function, and intellectual disability or developmental disorders. All SCZ patients were in a state of stable psychopathology and were taking neuroleptics, and some patients took antidepressants (*n* = 4), anxiolytics (*n* = 10), or mood stabilizers (*n* = 5). Among the anxiolytics, two patients were taking benzodiazepines (alprazolam and estazolam) and six patients were receiving clozapine at the time of EEG recording. Moreover, none of the patients were identified as treatment‐resistant based on clinical records, and all generally responded well to treatment. Participants were financially rewarded upon the completion of the research. The study had been approved by the Research Ethics Committee at the Institute of Psychology, Jagiellonian University in Krakow, Poland, and conducted following the ethical standards of the World Medical Association (World Medical Association 2013). The data from 5 participants (2 SCZ, 3 CON) were excluded due to poor quality of the EEG signal or excessive artifacts (Table 1).

**TABLE 1:** Demographic and clinical data.

	Schizophrenia group (*n* = 28)	Control group (*n* = 27)	Between‐group differences
Demographic	mean (SD)	mean (SD)	
Age	41.14 (8.87)	41.52 (8.01)	*t* = −0.16; ns
Sex: male/female	16/12	15/12	*X* ^2^ = 0.891; ns
Education (in years)	14.29 (2.62)	16.18 (2.73)	*t* = −2,63; *p* < 0.01
Work status (months last year)	6.21 (5.67)	10.67 (3.84)	*t* = −3.39; *p* < 0.001
MoCA	23.54 (3.42)	27.11 (1.89)	*t* = −4.78; *p* < 0.0001
**Clinical**			
*Characteristics of the illness*			
Duration of psychosis (in years)	16.89 (8.74)		
Number of relapses	8.79 (7.44)		
Number of hospitalizations	8.57 (5.66)		
Chlorpromazine equivalent (mg/day)	429.82 (283.88)		
*PANSS*			
Total	61.57 (15.36)		
Positive	11.36 (4.19)		
Negative	17.00 (6.30)		
Disorganisation	9.46 (3.97)		
Excitement	6.07 (2.26)		
Emotional distress	9.32 (3.13)		
*BNSS*			
Total	21.75 (12.65)		
Anhedonia	5.14 (3.88)		
Asociality	3.28 (2.37)		
Avolition	3.18 (2.18)		
Blunted effect	6.25 (4.26)		
Alogia	2.96 (2.47)		
*Schizophrenia ICD‐10*			
Paranoid (F20.0)	25 (91 %)		
Undifferentiated (F20.3)	2 (6 %)		
Schizoaffective disorder (F25.0)	1 (3 %)		
*Type of pharmacotherapy*			
Typical antipsychotics	1 (3%)		
Atypical antipsychotics	25 (91%)		
Typical and atypical antipsychotics mixed	2 (6%)		
Anxiolytics	10 (37%)		
Antidepressants	4 (14%)		
Mood stabilizers	5 (20%)		

*Note*: antidepressants: escitalopram, paroxetine; anxiolytics: alprazolam, estazolam, hydroxyzine; aripiprazole; atypical antipsychotics: amisulpride, clozapine, olanzapine, risperidone, sulpiride, quetiapine; Typical antipsychotics: flupentixol, haloperidol, promazine; mood stabilizers: carbamazepine, lithium, valproic acid. Subjects' demographics and clinical data were presented as mean (SD) for quantitative data and *n* (%) for the nominal variable. The significance level in all statistical analyses equaled alpha = 0.05.

Abbreviations: BNSS, Brief Negative Symptom Scale; ICD‐10, International Classification of Diseases, Tenth Revision; MoCA, Montreal Cognitive Assessment; PANSS, Positive and Negative Syndrome Scale.

### 2.2 Experimental Procedure

Resting‐state EEG recordings were conducted using the PsychoPy v1.82.01 software (Peirce 2008; Peirce et al. 2019). All participants in the scanning room were instructed to be relaxed and in a comfortable posture with minimal head movement during recording. They were to stare at a fixation cross presented for 300 s on the computer monitor.

### 2.3 EEG

#### 2.3.1 Recording

EEG data were obtained with a Biosemi Active Two amplifier system with 64 active electrodes positioned according to the standard 10–20 system and a sampling rate of 256 Hz. Four extra sensors were used to monitor oculomotor activity, and linked mastoids were used for the recording reference.

#### 2.3.2 Relative Power and ciPLV Preprocessing and Analysis

Data Preprocessing: Data were pre‐processed in MATLAB version 2021a with EEGLAB (The MathWorks, Inc.). The following sequence of steps was performed in order to remove artifactual (i.e., non‐cerebral) sources of electrical activity that may contaminate EEG recordings. First, EEG data was band‐pass filtered at 1–80 Hz. Next, the Zapline method was used to remove the top 6 components around the 50 Hz main line frequency (de Cheveigné 2020). Then, we removed bad channels using EEG lab's PREP plugin (Bigdely‐Shamlo et al. 2015) with default settings and interpolated the rejected channels. After which, Infomax ICA was performed and specific ICA components were rejected related to (i) eye blinks/movements using the EyeCatch algorithm default settings (Bigdely‐Shamlo et al. 2013), and (ii) muscle artifacts flagged by ICLabel at > 50% probability (Pion‐Tonachini et al. 2019). We then automatically removed additional low‐frequency artifacts using wavelet ICA at threshold = 10 and wavelet level = 10 (Castellanos and Makarov 2006). Finally, remaining EEG artifacts were removed epoch‐wise with a *z*‐score‐based method using the FASTER plug‐in (Nolan et al. 2010), rejecting 1‐s epochs deviating by more than two standard deviations. 1/f component was included in the relative power calculations.

Data Analysis: The cleaned EEG data was analyzed using the Neurophysiological Biomarker Toolbox (NBT; https://github.com/NBT‐Analytics/NBTpublic) implemented in MATLAB 2023a environment. The data were first re‐referenced to a standard average and later band‐pass filtered at 2–46 Hz, in order to reduce the power of any remaining low frequency (< 2 Hz, e.g. due to movement) and high‐frequency (> 46 Hz, e.g. due to line noise) artifacts. The power spectral density was computed across the five frequency bands of delta (2–3 Hz), theta (4–8 Hz), alpha (9–13 Hz), beta (14–30 Hz), and gamma (31–45 Hz) using the Welch method. The resulting relative power and ciPLV were calculated for each frequency band and electrode, separately for SCZ and CON, and then across all electrodes for group averages. Relative power is a metric that measures the power ratio of a frequency to the total power of all other frequencies. The ciPLV is a functional connectivity metric derived from comparing the phase consistency, or PLV, between two channels at a given time (Bruna et al. 2018). The ciPLV algorithm improves on the classical basic PLV by removing the contribution of zero phase differences in order to reduce biased estimates that are due to volume conduction (Bruna et al. 2018). In comparison, lagged coherence is sensitive to both phase and amplitude correlations between signals and thereby conflates these two mechanisms (Srinath and Ray 2014). PLV specifically focuses on phase synchronization, which is thought to be crucial for neuronal communication through the alignment of temporal “excitability windows” (Fries 2015).

To compute node strength, the averages for each of the 64 electrodes for the SCZ and CON were computed from the pairwise connectivity values.

### 2.4 Statistical Analysis

Differences in relative power and ciPLV between groups were assessed using non‐parametric permutation tests with cluster‐based correction via NBT, with significance determined from 50,000 permutations (Meyer et al. 2021). Since the permutation distribution is strictly data‐driven and nonparametric, no degrees of freedom are provided. To explore potential associations between EEG measures (relative power and ciPLV) and clinical variables, we conducted correlation analyses. The clinical variables included PANSS total scores, PANSS subscale scores (Positive, Negative, General Psychopathology), BNSS total scores, duration of illness, and medication dosage expressed as chlorpromazine equivalents. Due to the non‐parametric nature of the data and the small sample size, we used Spearman's rank‐order correlation (Spearman's rho) and Kendall's tau‐b correlation coefficients for these analyses. Effect sizes were quantified using Cohen's *d*, thereby assuming effects to be small (*d* = 0.2), moderate (*d* = 0.5), and large (*d* = 0.8). Outliers were detected by the interquartile range (IQR). Multiple comparisons were FDR‐corrected (Korthauer et al. 2019), with a significant value threshold of *p* < 0.05

## 3 Results

### 3.1 Relative Power Spectrum Density

**FIGURE 1:**
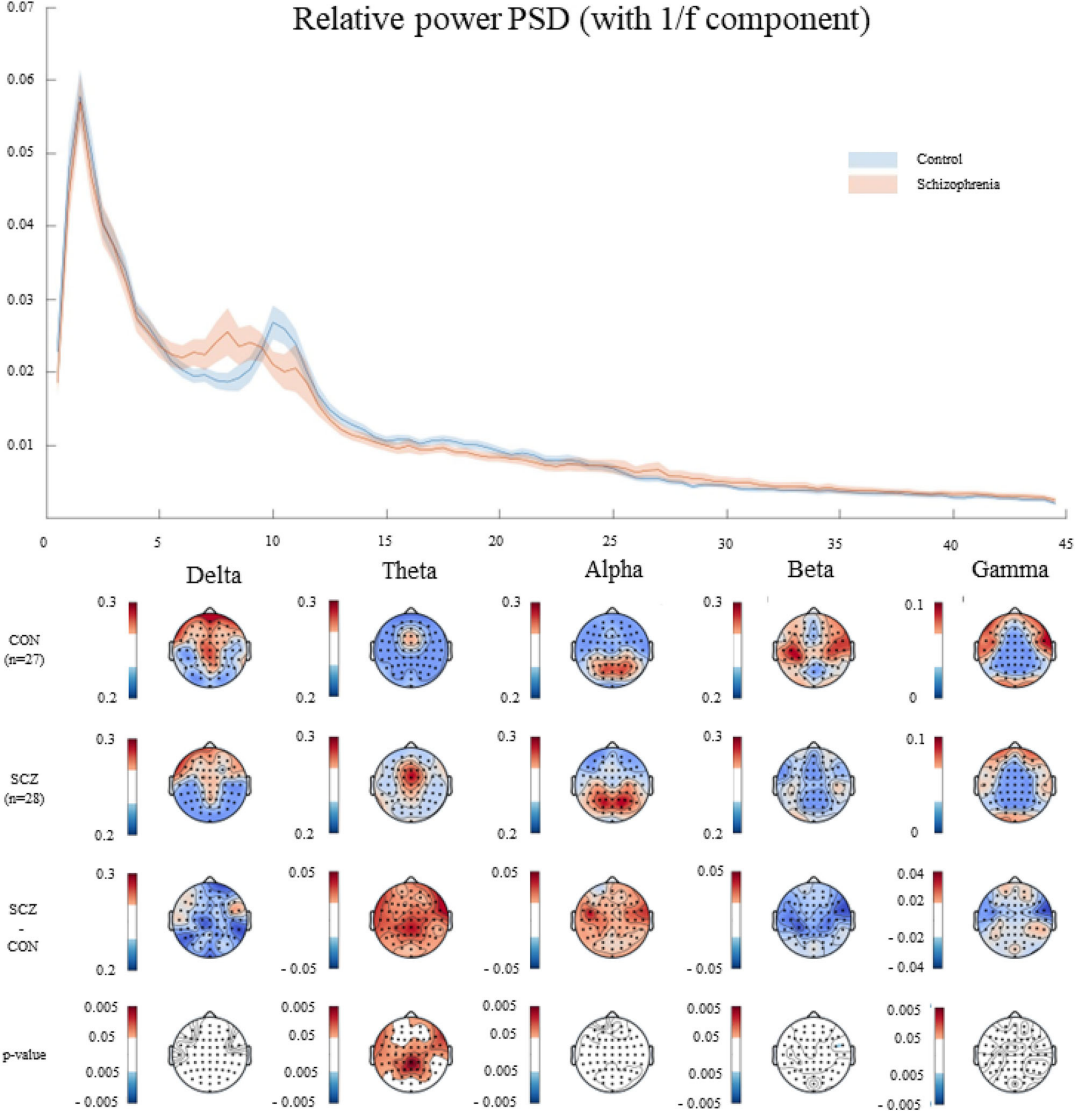
(Top figure) Broadband relative power spectrum: the *y*‐axis represents the relative power spectral density (PSD), and the *x*‐axis the brain waves frequencies in Hz; (Bottom figure) Topographic maps concerning the broadband relative power differences within‐group and in the last two rows the between‐group comparisons. The red indicates the increased values at SCZ > CON, and the blue at CON > SCZ contrasts.

The relative power spectrum analysis revealed distinct patterns between the SCZ group and healthy controls. Specifically, the SCZ group showed a notable peak within the theta frequency band, ranging from 5.5 to 9.5 Hz, with a pronounced peak at approximately 8 Hz. Conversely, the peak for the CON group was identified at around 10 Hz (Figure 1).

The most significant between‐group differences in relative power in theta band (SCZ > CON) were observed across several brain regions. These included frontal areas: F8 (*d* = 0.84; *p* = 0.008), FT8 (*d* = 0.91; *p* = 0.004), FC6 (*d* = 0.84; *p* = 0.008); central regions: CP3 (*d* = 0.89; *p* = 0.005), CP1 (*d* = 0.92; *p* = 0.005), Cz (*d* = 0.86; *p* = 0.008), C2 (*d* = 0.81; *p* = 0.008), C4 (*d* = 0.81; *p* = 0.009), CP2 (d = 0.97; *p* = 0.005); and parietal lobes: P1 (*d* = 0.95; *p* = 0.004), Pz (*d* = 1.07; *p* = 0.002), CPz (*d* = 1.09; *p* = 0.002), P2 (*d* = 1.04; *p* = 0.004). Only Cohen *d* effect sizes > 0.8 are reported (detailed in Supporting Materials).

### 3.2 Corrected Imaginary Phase‐Locking Value (ciPLV)

**FIGURE 2:**
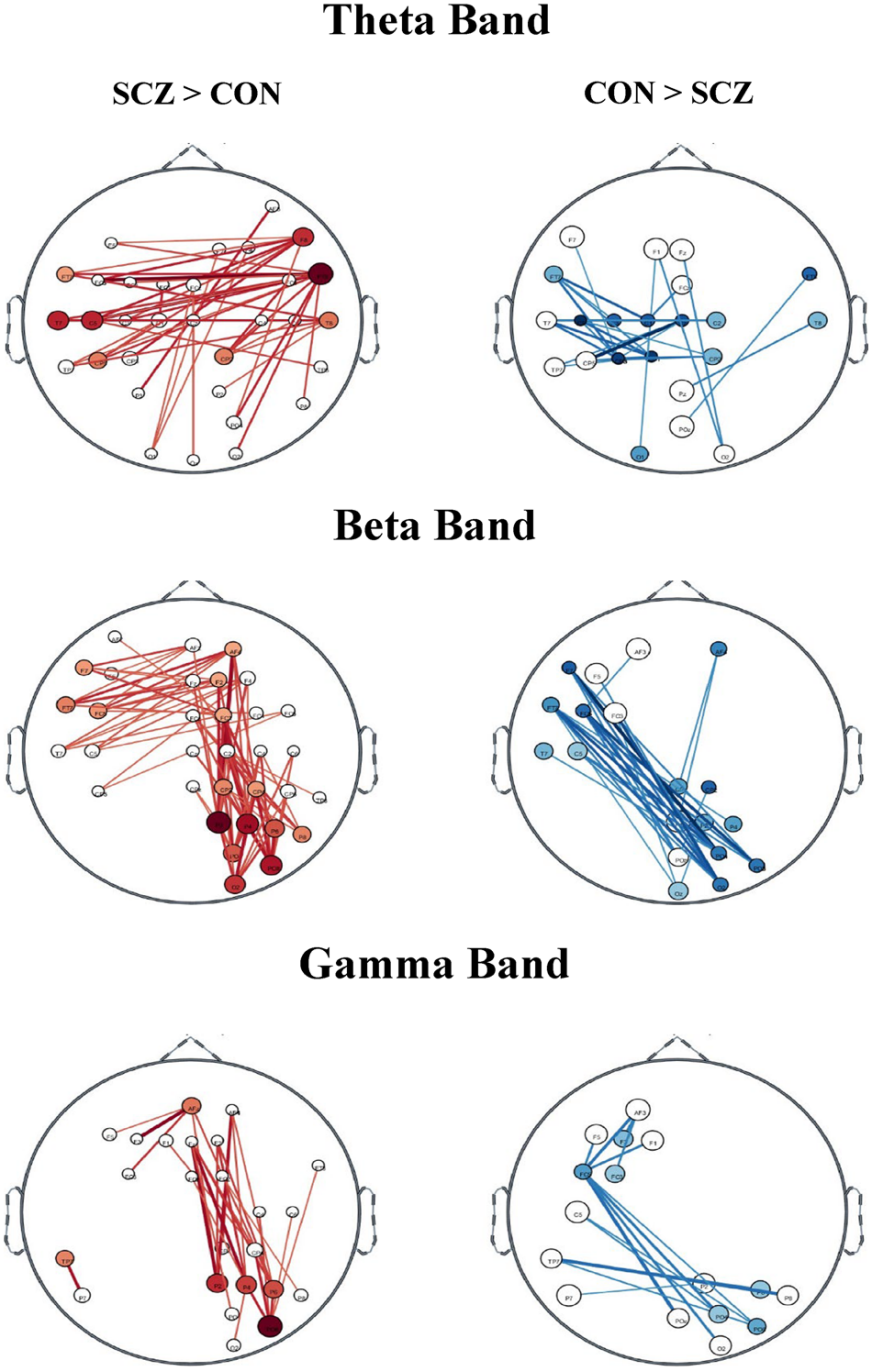
Comparison of ciPLV‐based functional connectivity between individuals with chronic schizophrenia (SCZ) and healthy controls (CON) across 64 scalp electrodes. Red lines indicate significantly stronger connectivity in SCZ relative to CON, while blue lines indicate stronger connectivity in CON relative to SCZ. Node positions correspond to electrode locations, and line thickness reflects the magnitude of connectivity differences. Frequency‐specific results are presented in separate panels (theta, beta, gamma), illustrating distinct connectivity patterns between groups.

The ciPLV analysis underscored significant differences in connectivity patterns between the SCZ and CON groups, highlighting variations in the strength and number of connections, as well as divergent inter‐ and intra‐hemispheric patterns across theta, beta, and gamma frequency bands (refer to Figure 2 and Supporting Materials).

In the theta band, enhanced inter‐hemispheric connectivity was predominantly observed in the SCZ group, whereas the CON group exhibited mainly intra‐hemispheric hyper‐connectivity within the left hemisphere Notably, nodes such as FT8 and F8 (*d* > 1.00; *p* < 0.05 FDR corrected) in the SCZ group demonstrated a more dispersed or widespread inter‐hemispheric connectivity pattern, with distinct differences in the connectivity patterns observed in channels like CP2 across the two groups. In the wider context, our findings of specifically increased interhemispheric connectivity in the theta band, do not support the popular narrative of SCZ being a syndrome of reduced connectivity or “disconnectivity” (Pettersson‐Yeo et al. 2011).

Beta band results illustrated an inverse pattern to the theta band, with the SCZ group showing primarily intra‐hemispheric hyper‐connectivity, especially among frontoparietal nodes, and reduced inter‐hemispheric frontotemporal connections. The CON group, in contrast, displayed pronounced inter‐hemispheric connectivity. Channels such as AF4, FT7, P2, P4, and PO8 (*d* > 1.10; *p* < 0.05 FDR corrected) in the SCZ group, and F7, FC5, FT7, PO4, PO8, and O2 (*d* > 1.00; *p* < 0.05 FDR corrected) in the CON group were among those with significant connectivity differences, indicating varied interaction directions and strengths between shared nodes (e.g., AF4 ‐ more interactions for the SCZ group (*d* > 1.10; *p* < 0.05 FDR corrected), while its connectivity is minimal in the CON group (*d* < 0.85; *p* < 0.05 FDR corrected).

Although the connectivity differences were weaker in the gamma frequency band, they resembled those observed in the beta band. Here, the most hyper‐connected channels were PO8 (*d* > 1.00; *p* < 0.05 FDR corrected) for the SCZ group and FC5 (*d* > 1.00; *p* < 0.05 FDR corrected) for the CON group.

In the delta and alpha frequency bands, the ciPLV analyses did not reveal any significant differences in functional connectivity patterns between the SCZ and CON groups. After applying FDR correction for multiple comparisons, no significant connectivity differences were found in these bands across any electrode pairs (all *p*‐values > 0.05).

### 3.3 EEG Measures and Their Association With Clinical Variables

Spearman's rho and Kendall's tau–b correlation analyses were performed to obtain a measure of association between EEG measures and clinical variables in the SCZ group. The measures of EEG included both relative power in each frequency band and ciPLV values. Clinical variables analyzed included PANSS total score, PANSS subscale scores (Positive, Negative, General Psychopathology), BNSS total score, duration of illness, and medication dosage (chlorpromazine equivalents). No significant correlations were found between EEG and clinical variable measures (*p* > 0.05).

## 4 Discussion

This investigation advances our understanding of SCZ's neurophysiological nuances by examining a new, volume‐conduction‐corrected version of ciPLV as a potential clinical biomarker. To the best of our knowledge, this study provides the first set of data on the ciPLV functional connectivity metric of resting‐state‐EEG observed in patients with SCZ. Our key findings indicate that patients with SCZ exhibited significantly increased theta band (4–8 Hz) relative power in centroparietal regions, specifically at channels CPz, Pz, and P2, compared to healthy controls. In addition, we observed altered functional connectivity patterns characterized by increased inter‐hemispheric connectivity in the theta band and widespread bilateral changes across multiple frequency bands. Notably, there was a reversal of hemispheric activation patterns in the theta and beta/gamma bands when compared to controls. However, no significant differences were found in delta and alpha power after FDR correction in connectivity, and we did not identify significant associations between EEG measures and clinical variables such as PANSS scores, BNSS scores, duration of illness, or medication dosage. These findings suggest that the EEG alterations may represent trait‐related abnormalities in SCZ, providing new insights into the neurobiological mechanisms underlying the disorder.

### 4.1 Relative‐Power Spectrum Characteristics of SCZ

The study confirms previous statements on the role of theta (low‐frequency) band as a crucial marker in SCZ, corresponding to already‐established viewpoints (Cao et al. 2022; Iglesias‐Tejedor et al. 2022; Newson and Thiagarajan 2018). In this respect, our research differs in the analysis of higher‐frequency bands, where the reported differences were not significant after the FDR correction (Cao et al. 2022; Iglesias‐Tejedor et al. 2022; Newson and Thiagarajan 2018). Moreover, increased theta oscillations were mainly seen in the centroparietal region (the CPz, Pz, and P2 channels), which could reflect activity from the superior parietal cortex and precuneus (Koessler et al. 2009). In addition, consistent increases in theta activity were observed bilaterally in the frontotemporal regions. The pattern of increased power of this low frequency is also present in other psychiatric conditions, such as ADHD or OCD (Newson and Thiagarajan 2018), pointing to a possible transdiagnostic marker of cognitive dysfunction (McLoughlin et al. 2022).

Previous research indicates a number of resting‐state EEG signatures in SCZ, with some studies showing reduced alpha and beta bands linked to the diagnosis of the disorder, its symptomatology, or chronic nature (Knyazeva et al. 2008; Sponheim et al. 2000). It has been proposed that the front‐beta and gamma oscillations during the eyes‐open resting state might represent a unique or prognostic marker that can be consistently observed among SCZ patients and their relatives (Venables et al. 2009). In addition, other research suggests that gamma‐band activity could relate to the manifestations of psychotic symptoms (Yadav et al. 2021), especially in unmedicated patients (Shin et al. 2011), with significant task‐related increases, which are not found during the eyes‐open resting state (Hirano et al. 2015)—the latter mirroring our findings.

The lack of significant beta and gamma power differences in our study could be explained by factors such as sample size, illness stage, symptom severity, and/or type of medication. Considering this, our findings reinforce the presence of excessive theta oscillations between 4–8 Hz in centroparietal regions during eyes‐open states in chronic schizophrenic patients in stable conditions and under long‐term medication. To advance further, future studies will be needed to separate the role and specificity of power‐spectrum biomarkers in SCZ. This could eventually help in refining diagnostic and therapeutic strategies.

### 4.2 Differences in Functional Connectivity Using Corrected Imaginary Phase‐Locking (ciPLV)

This section of our study delves into the topographic differences in functional connectivity, as quantified by ciPLV metrics, presenting a novel analysis within the context of SCZ.

In contrast to the historical literature, our study did not show any pronounced differences in delta band connectivity, which is in contradistinction with previous studies that noted highly hyper‐connected left frontal region and its widespread brain connections (di Lorenzo et al. 2015; Kam et al. 2013; Tauscher et al. 1998).

Our connectivity findings within the theta band are in line with those of Andreou, Leicht, et al. (2015) and Kam et al. (2013) who reported increased inter‐hemispheric connectivity between parietal areas. Nevertheless, a considerable difference from previous studies is the observation of widespread frontal hyper‐connectivity, extending beyond the predominantly left‐hemisphere hyper‐connectivity documented in prior research (di Lorenzo et al. 2015; Tauscher et al. 1998). Consistent with this, di Lorenzo et al. (2015) reported that the theta band had excessive hyper‐connectivity throughout the brain without implicating specific nodes (both intra‐ and inter‐hemispheric). Lastly, Winterer et al. (2001) revealed hypo‐connectivity in the temporal lobes, contrary to our findings of hyper‐connectivity.

The study failed to reveal a significant reduction in alpha band connectivity, in contrast to claims of decreased coupling in this band (di Lorenzo et al. 2015; Kam et al. 2013; Tauscher et al. 1998). Notwithstanding, the same authors reported increased intra‐hemispheric connectivity within the beta band, which we also observed, particularly in the right hemisphere. Our study adds additional nuances by identifying a cross‐hemispheric hypo‐connectivity pattern in this frequency band.

Furthermore, our gamma band results reveal increased connectivity, particularly within right frontoparietal regions in SCZ patients, partially corroborating findings by Kikuchi et al. (2011), which noted disrupted connectivity in the right frontal region. It is worth mentioning that in that study, participants with SCZ were medication‐naïve, while in our study, they were under antipsychotic medication. Moreover, other studies pinpointed intra‐hemispheric hyper‐connectivity and the connection pattern of frontal‐posterior offer nearly the same results, indicating reduced communication within the left hemisphere in SCZ (Andreou, Nolte, et al. 2015; Baradits et al. 2019). In support, similar results were found in the connectivity pattern in the right hemisphere (di Lorenzo et al. 2015), although we also report some hypo‐connectivity (CON > SCZ) patterns, especially in the left frontal area nodes.

The central theme emerging from our ciPLV analysis seems to be the finding of widespread bilateral changes in neural communication extending across multiple frequency bands. The most significant difference exhibited by the CON and the SCZ groups was manifested in the reversal of hemispheric activation patterns in the theta and beta/gamma bands. Taking into account the different findings that are reported in the literature, our study thus highlights the need for more extensive investigations to be conducted with great detail in order to explain the complex core metrics of ciPLV within the SCZ spectrum, which, in turn, could advance our understanding of the neurobiological mechanisms underlying psychopathology.

### 4.3 Associations Between EEG Measures and Clinical Variables

Despite the significant differences observed in EEG measures between the SCZ and CON groups, our correlation analyses did not find significant associations between EEG measures and clinical variables such as PANSS scores, BNSS scores, duration of illness, or medication dosage. This lack of significant correlations suggests that the EEG alterations may represent trait‐related abnormalities in SCZ rather than state‐dependent changes related to symptom severity.

### 4.4 Limitations and Future Directions

Despite providing valuable insights into the neurophysiological characteristics of SCZ, this study has several limitations. The relatively small sample size may have reduced the statistical power to detect significant associations between EEG measures and clinical variables, limiting the generalizability of our findings. In addition, two patients (7.1%) were taking benzodiazepines, and six patients (21.4%) were on clozapine—medications known to affect EEG activity—which could have influenced the results. While the proportion of patients on benzodiazepines is less than 20% and thus unlikely to drive group differences, the inclusion of patients on clozapine raises the percentage of those on medications with sedative properties to 28.6%. However, clozapine is a standard antipsychotic treatment for SCZ, and its sedative effects are generally less pronounced and diminish over time. The sample size was insufficient to analyze the specific impact of these medications separately. The cross‐sectional design also limits our ability to infer causal relationships between EEG alterations and symptomatology.

Future research should address these limitations by recruiting more diverse samples to enhance statistical power and generalizability. Controlling for medication effects or including medication use as a covariate in analyses would help clarify the influence of medications like benzodiazepines and clozapine on EEG measures. Longitudinal studies are needed to examine how EEG connectivity patterns change and relate to symptom progression or treatment outcomes. Incorporating comprehensive cognitive and functional assessments could provide a more holistic understanding of how EEG alterations relate to clinical features of SCZ.

## 5 Conclusions

The results of our analyses shed light on the electrophysiological abnormalities characterizing SCZ, focusing on FNC patterns in sensor space. Principally, we observed theta and beta/gamma connectivity deviations between fronto‐temporo‐parietal areas. Changes in these connections could be potential neural markers of SCZ. The uncovered EEG abnormalities may be informative as “candidate” targets for neuromodulation treatments (e.g., tACS or EEG neurofeedback), which could then confirm or reject the causal involvement of these markers in SCZ symptomatology. However, whether they precede or result from long‐term pharmacotherapy remains uncertain. The outcome of the ciPLV analyses both confirmed and contradicted previous FNC findings in SCZ. Hence, they may inform the debate regarding heterogeneous FNC signatures of SCZ in the literature. Finally, local power abnormalities in the theta band reinforce the clinical significance of oscillations in this frequency, replicating their critical role in SCZ.

## Author Contributions

**Christophe Domingos**: methodology, formal analysis, software, validation, visualization, writing – original draft. **Wiktor Więcławski**: validation, writing – review and editing. **Sandra Frycz**: writing – review and editing. **Maja Wojcik**: writing – review and editing. **Martin Jáni**: investigation. **Olga Dudzińska**: investigation. **Przemysław Adamczyk**: conceptualization, funding acquisition, project administration, methodology, resources, investigation, supervision, validation, writing – review and editing. **Tomas Ros**: conceptualization, data curation, software, resources, supervision, writing – review and editing.

## Ethics Statement

All procedures performed in studies involving human participants were in accordance with the ethical standards of the institutional, national research committee (The Research Ethics Committee at the Institute of Psychology, Jagiellonian University KE/02/082017) and with the 2013 World Medical Association Declaration of Helsinki (2013).

## Consent

Informed consent was obtained from all participants in the study.

## Conflicts of Interest

The authors declare no conflicts of interest.

### Peer Review

The peer review history for this article is available at https://publons.com/publon/10.1002/brb3.70370

## Supporting information



Statistical analysis of EEG channels, including P‐values and effect size (Cohen's D) for the differences between subject groups

Statistical analysis of EEG channels, including P‐values and effect size (Cohen's D) for the differences between subject groups

## Data Availability

The data that support the findings of this study are available from the corresponding author upon reasonable request.
